# Occurrence, Diversity, and Character of *Bacillaceae* in the Solid Fermentation Process of Strong Aromatic Liquors

**DOI:** 10.3389/fmicb.2021.811788

**Published:** 2022-01-31

**Authors:** Wenhua Tong, Ping He, Ying Yang, Zongwei Qiao, Dan Huang, Huibo Luo, Xinjun Feng

**Affiliations:** ^1^College of Bioengineering, Sichuan University of Science and Engineering, Yibin, China; ^2^CAS Key Laboratory of Biobased Materials, Qingdao Institute of Bioenergy and Bioprocess Technology, Chinese Academy of Sciences (CAS), Qingdao, China; ^3^Wuliangye Yibin Co. Ltd., Yibin, China

**Keywords:** strong aromatic *Baijiu*, *Bacillaceae*, diversity, function, flavor

## Abstract

Strong aromatic liquors, also known as strong aromatic *Baijiu* (SAB) in China, are manufactured by solid fermentation, with a multi-microbe mixing and cooperative fermentation process that uses *Daqu* as a brewing starter. *Bacillaceae* have a specific action in food fermentation, such as soybean and wine, and more recent studies have found *Bacillaceae* play important roles in the SAB making industry. This review describes the diversity, functionality, and influence of *Bacillaceae* in *Daqu*, pit mud, *Zaopei*, *Huangshui* within making processes of SAB. Furthermore, aromatic flavor components from the *Bacillaceae* metabolism of SAB are discussed in this review. Ultimately, the resulting improvements and deeper understanding will benefit practical efforts to apply representatives of *Bacillaceae* in improving the quality of SAB as well as biological control of the micro-ecological environment of brewing.

## Introduction

**FIGURE 1:**
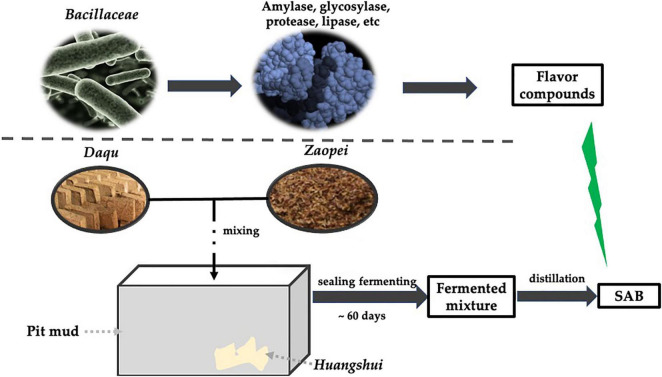
*Bacillaceae* associated with the process of SAB brewing.

*Baijiu* is a traditional distilling liquor made by solid fermentation that has been made for generations, stretching back over a 100 years. According to the different flavors, *Baijiu* can be divided into three most basic types (strong aromatic-flavor, sauce-flavor, light flavor) and nine derived types from above the three flavors (rice-flavor, jian-flavor, fuyu-flavor, te-flavor, feng-flavor, dong-flavor, chi-flavor, sesame-flavor, laobaigan-flavor) (Zheng and Han, 2016). SAB is world famous for its special flavor, occupying about 70% *Baijiu* market in China, Wuliangye is the representative brand of SAB, and the annual production of SAB exceeded 10 million tons (Liu et al., 2017b; Xu et al., 2017). SAB is mainly made from multiple grains (sorghum, corn, rice, wheat, glutinous rice, and so on), with *Daqu* as a primary saccharification starter, and a solid state fermentation process was carried out in a fermented mud pit. Ultimately, alcohol and various aromatic materials are obtained by distilling (Figure 1). The production of SAB was carried out in 60 days or so, and complex metabolic reactions were detected during the long-term SAB fermentation (Hu et al., 2021b). Subsequently, the physical and chemical characteristics of *Zaopei* (mixture of steamed grains, rice husks, fermented grains, etc.) changed constantly, as a result, abundant compounds were produced (Guan et al., 2020), which were closely associated with the style of SAB. Owing to the natural fermentation style, numerous microbes are involved in the SAB making process (bacteria and fungi) (Ma et al., 2016; Wang et al., 2017; Zhang et al., 2017; Liu et al., 2018b; Qian et al., 2021), which mainly originated from *Daqu*, pit mud, the environment of the distillery and so on. The final SAB liquor structure is produced by the coaction of these microbes (Hu X. et al., 2016). Until now, studies on the microbes of SAB brewing focused on the microbial community structure and relevant flavor substances. Zou et al. (2018) discussed the diversity and function of the microbial community in SAB at the macro level. Several fungi and bacteria were are discussed in this work, however, a detailed introduction of the role for specific species of microbes throughout the SAB ecosystem is lacking, which is vital for brewing quality control. As important food-associated microorganisms, especially in SAB production, the function and roles of *Bacillaceae* were elaborated in the major fermentation phase, thus research in this field will benefit from SAB production by bioaugmentation (or other regulation strategies) of *Bacillaceae* in *Daqu*, pit mud, *Zaopei*, and *Haungshui* (a brown liquid byproduct was formed during SAB brewing, and deposited in the pit bottom) (Ding et al., 2015).

*Bacillaceae* is a gram-positive bacteria that plays an important role in the SAB making industry (Zheng et al., 2011; Ding et al., 2014; Wang et al., 2014; He et al., 2019b). Most of the *Bacillaceae* isolated from *Daqu*, pit mud, *Zaopei*, and *Haungshui* can produce various aromatic flavor components or flavor precursor, such as acetoin, 2,3-butanediol, four-carbon compound, pyrazines, and so on (Liu et al., 2017c; Zhang et al., 2021a). This feature could also be observed in the making process of various fermented foods. It is well known that the principal fragrance component is ethyl caproate in SAB which is associated with caproic acid bacteria (Luo et al., 2020; Cheng T. et al., 2021). Caproic acid bacteria is a group of microorganisms that can metabolize caproic acid, which mainly consists of *Bacillus megaterium*, *Bacillus fusiformis*, *Bacillus licheniformis*, and *Clostridium*, etc. (Zhao et al., 2012; Hu et al., 2015). Wang et al. (2015b) identified eight *Bacillus* from *Daqu* by employing the traditional method. The results showed that they belonged to five specials, *Bacillus megaterium*, *Paenibacillus macerans*, *Bacillus pumilus*, *Bacillus atrophaeus*, and *Bacillus licheniformis*, respectively. Liu et al. (2017c) identified three dominant *Bacillus* from a century mud pit of Luzhou Laojiao Liquor distillery, which showed *Lysinibacillus sphaerieus*, *Brevibacillus brevis*, and *Paenibacillus larvae subsup. Pulvifaciens*. The distribution *of Bacillaceae* have significant differences among *Daqu*, pit mud, *Zaopei*, and *Huangshui* in different geographical environments and seasons. A previous study suggested that the temperature variation and humidity may be important factors affecting the microbe community (Fu et al., 2011; Yin et al., 2014), as there are differences in these factors in various SAB distilleries (Table 1).

**TABLE 1:** Difference distribution of *Bacillaceae* in SAB distilleries producing *Daqu.*

Sample	Places	Representative strains	References
*Daqu* of “Luzhou Laojiao”	Luzhou City, Sichuan Province	*Bacillus subtilis*, *Bacillus licheniformis*	Yao et al., 2005
*Daqu* of “Wuliangye”	Yibin City, Sichuan Province	*Bacillus subtilis*, *Bacillus cereus*	Zhao et al., 2009, Liang et al., 2017, Fan et al., 2021
*Daqu* of “Gujingong”	Haozhou City, Anhui Province	*Bacillus subtilis, Bacillus licheniformis, Bacillus amyloliquefaciens, Paenibacillus Castaneae, Bacillus pumilus, Bacillus subtilis SS Subtilis, Bacillus circosus, Bacillus cereus*, etc.	Liang et al., 2017
*Daqu* of “Yingjia Gongjiu”	Liuan city, Anhui Province	*Bacillus pumilus, Bacillus amyloliquefaciens, Bacillus licheniformis, Paenibacillus nicotianae*	Wang et al., 2021
pit mud of “Luzhou Laojiao”	Luzhou City, Sichuan Province	*Lysinibacillus sphaerieus, Brevibacillus brevis, Paenibacillus larvae subsup. pulvifaciens*	Liu et al., 2017c
pit mud of “Yanghe Daqu”	Suqian City, Jiangsu Province	*Clostridium Kluyverii, Ruminiclostridium (Clostridium tyrobutyricum, Clostridium butyricum)*	Wang et al., 2015a, Gou et al., 2020
pit mud of “Gujingong”	Haozhou City, Anhui Province	*Bacillus licheniformis, Bacillus coagulans, Bacillus amyloliquefaciens, Bacillus atrophus, Bacillus mohighi, Bacillus pumilus*	Wu et al., 2016
pit mud of “Zhijiang”	Zhijiang City, Hubei Province	*Bacillus, Sporolactobacillus*	You et al., 2009
pit mud of “Daohuaxiang”	Yichang City, Hubei Province	*Clostridium*	Wang et al., 2010
pit mud of “Jiannanchun”	Mianzhu City, Sichuan Province	*Bacillus, Clostridium* (*Clostridium sporogense, Clostridium aminophilum, Clostridium cochlearum, Clostridium innocuum*)	Liu et al., 2017c, Xu Z. et al., 2019
*Zaopei* of “Xufu”	Yibin City, Sichuan Province	*Bacillus subtilis, Bacillus amyloliquefaciens, Bacillus drentensis, Bacillus stratosphericus, Bacillus anthracis, Bacillus safensis, Bacillus cereus, Bacillus vallismortis, Lysinibacillus fusiformis, Rummeliibacillus pycnu, Brevibacillus borstelensis*	Zhou et al., 2010
*Huangshui of “*Shuijingfang”	Chengdu city, Sichuan Province	Clostridia *(such as Clostridium_sensu_stricto, norank_f_Clostridiaceae)*, Bacilli	Gao Z. et al., 2020

As the dominant microorganism in the SAB making process, the review described the diversity of *Bacillaceae*, the function of *Bacillaceae* in *Daqu*, pit mud, *Zaopei*, and *Huangshui*. The effect on the flavor of SAB that contributes to *Bacillaceae* is also discussed. Through the analysis of this article, we hope to provide ideas for exploring the functional microorganisms in the SAB making industry, and accelerate a deeper understanding of the brewing mechanism of SAB, helping to improve the quality of SAB, and facilitating the better application of *Bacillaceae* in the food fermentation industry.

## Function of *Bacillaceae* in SAB Brewing Process

### *Bacillaceae* in *Daqu*

The volatile flavor characteristics of SAB are influenced by the microflora of raw materials and the environment. In the production of SAB, pit mud is the foundation of fermentation (Zheng et al., 2013; Zheng and Han, 2016), *Daqu* is the motive power of fermentation (Gou et al., 2015; Deng et al., 2020), and brewing technology is the core of fermentation. Numerous studies have shown that *Bacillus* contributes greatly to the flavor of SAB in *Daqu*, the secondary metabolites of *Bacillus* could interact with other microorganisms, and benefited from maintaining relative stability of the microecosystem of *Daqu* (He et al., 2019a; He G. et al., 2020). *Daqu*, as the saccharification starter of SAB, includes lots of microbes and enzymes, and *Bacillaceae* play a key role in the production of *Daqu* (Li et al., 2014; Hu et al., 2020; Xiang et al., 2020). For example, as the functional microbes in *Daqu*, *Bacillus licheniformis* could produce multiple enzymes, such as amylase, proteases, and lipases in *Daqu* (Yan et al., 2013b), besides, *Bacillus licheniformis* could also reduce the content of higher alcohols in SAB (too high to spoilt SAB taste, easy to cause headaches, easy to drunk) (Shi et al., 2012). *Bacillus* spp. could hydrolyze proteins in fermented foodstuffs, which benefits the flavor and flavor precursor formation in *Daqu* (Beaumont, 2002). Meanwhile, *Bacillus* spp. can also exhibit the capacity of inhibiting the growth of *Streptomyces*, and reduce geosmin to avoid soil odor in *Daqu* (Zhi et al., 2016). Zhai et al. (2020) analyzed. *Daqu* samples in the main product area of SAB (Yibin city) by high throughput sequencing, the result showed that microorganisms consist of *Bacillus* sp., lactic acid bacteria, *Pedicoccus sp.*, *Weissella* sp., *Leuconostoc* sp., *Thermoactinomyces* sp. and *Acetobacter* sp.). Research has shown that *Bacillus subtilis*, *Bacillus horneckiae, Bacillus megaterium*, *Bacillus licheniformis*, and *Brevibacillus* make up common species of *Bacillaceae* in *Daqu* (Zhou et al., 2010; Xiang et al., 2020).

The functions of *Bacillaceae* within *Daqu* in SAB brewing were as follows: firstly, various hydrolases were secreted into *Daqu*, and the utilization ratio of raw material was elevated (Wang et al., 2017; Wu et al., 2020); secondly, flavor substances were produced, for example, *Bacillus* spp. could produce abundant organic acids in *Daqu* (e.g., malic, lactic, acetic, citric, succinic, propionic and butyric acids), and the organic acids were the main flavoring components of SAB and the precursors of ester production (Yan et al., 2013a), meanwhile, the study also found that *Bacillus* spp. could produce aromatic substances in *Daqu* (benzaldehyde, 4-methylphenol, benzeneethanol, ethyl phenylacetate, etc.) (He et al., 2019b). Thirdly, the study revealed ingredients that are good for health can be enriched in *Daqu*, for example, *Bacillus licheniformis* directly proliferated in *Daqu*, which could significantly increase the content of pyrazines, terpene, and zearin, etc. (Zhang R. et al., 2013; Wang et al., 2015b; Zhou et al., 2016), substances that are good for human health (i.e., anti-cancer, anti-virus, anti-inflammatory, anti-oxidation). Recently, two strains of high yield of fibrinolysin (function as anti-thrombus) were also identified from *Daqu* (*Bacillus velezensis, Bacillus licheniformis*) (Zhang et al., 2021b). Fourthly, promoting the distillation of flavor components from *Zaopei*, and reducing the harmful components of microbial metabolite, *Bacillus licheniformis* could produce lichenin to enhance the volatilization of esters and other substances, and markedly decrease the volatilization of phenol and phenylethanol (Wang et al., 2015b).

### *Bacillaceae* in Pit Mud

The SAB fermentation process was carried out in a pit cellar, in which mud functioned as a fermentation carrier of SAB. Pit mud contains a variety of important microorganisms (Tao et al., 2014; Liang et al., 2015; Hu X. et al., 2016; Liu et al., 2017a), and the special pit aroma in SAB is mainly derived from pit mud microbes (Zhang et al., 2017). The bacteria in pit mud consist of *Bacillus, Clostridium, Pseudomonas, Sporolactobacillus, Lactobacillus*, and *actinomycetes*, etc. (Yue et al., 2007; Zou et al., 2018; Liu M. et al., 2020; Liang et al., 2021). PCR-DGGE analysis revealed that eight families of bacteria species existed in pit mud, e.g., *Clostridiaceae*, *Lactobacillaceae*, *Synergistaceae, Sphingomonadaceae, Ruminococcaceae, Clostridiales_*Incertae Sedis XI, *Lanchnospiraceae*, and *Plannococcaceae*. They belong to three main microorganisms of *Clostridiales, Lacotobacillaceae*, and *Bacillale*, separately (Zheng et al., 2013). Fifteen *Clostridium* species and one *Bacillus* were isolated from one of the best-known brands of SAB pit mud (Luzhou Lao Jiao liquor), all of them had the ability to produce organic acid (Guo et al., 2020). Recently, more bacteria of the *Bacillaceae* species were isolated from pit mud (e.g., *Aneurinibacillus migulanus*, *Bacillus pumilus*, *Lysinibacillus boronitolerans*, *Bacillus badius*, *Bacillus coagulans, Bacillus aerius, Bacillus subtilis, Bacillus licheniformis, Lysinibacillus sphaericus, Bacillus pulvifaciens, Bacillus pumilus*, *Virgibacillus pantothenticus, Sporolactobacillus, Brevibacillus* sp*., Bacillus sphaericus, Bacillus niabensis, Bacillus bataviensis, Bacillus cereus*) (Zhang et al., 2010, 2019; Liu et al., 2013, 2017c, 2018c; Ye et al., 2013; Wang et al., 2018). The culturable bacteria’s from pit mud were *Bacillus* species, and the predominant strains were *Bacillus licheniformis* and *Bacillus subtilis* (Zhang et al., 2010). Most of the *Bacillaceae* isolated from pit mud can produce acetoin, 2,3-butanediol, C4 compounds, pyrazines, volatile acid, etc., all the compounds listed above are important flavors, and are crucial to regulating the SAB flavor (Wu et al., 2019; Table 2).

**TABLE 2:** Main flavors produced by identified SAB *Bacillaceae* in various fermentation processes.

*Bacillaceae*	Substrate	Category	Test method	References
*Bacillus subtilis*	Soymilk/sorghum/corn fermentation	**Hydrocarbons** (nonane, 5-methyl-1-heptene, 2,6,10-trimethyldodecane, 1,3-dimethylnaphthalene, 2,3-dimethylnaphthalene); **acids** (acetic acid, 2-methyl-propanoic acid, propanoic acid, butanoic acid, 3-mehtyl butanoic acid, 2-methyl butanoic acid, glyoxylic acid, tryptophan. lysine, leucine, isoleucine, aspartate, phenylalanine, valine, histidine, methionine, alanine, tyrosine, glutamate, glycine, taurine, γ-aminobutyrate, 6-phosphogluconic acid, lactate, succinate, pyruvate, fumarate, malonate, citrate, isobutyrate, isovalerate, 2-methylbutyrate, 2-hydroxyisobutyrate, 3-hydroxybutyrate, β-hydroxyphenylacetate, α-ketoisovalerate); **alcohols** (ethanol, isopropyl alcohol, butanol, pentanol, 2,3-butanediol, 1,3-butanediol, 2-butanol, β-phenylethanol, n-butanol, cedrol, phenylethyl alcohol, isooctanol, 2-(1-methoxyethoxy)ethanol, 1,2-propanediol, methanol, isopropanol); **aldoketones** (butanedione (diacetyl), 2,3-butanedione, 3-hydroxy-2-butanone, 5-hydroxy-4-octanone, benzeneacetaldehyde, acetoin, 2-heptanone, 2-non-anone); **esters** (butanoic acid butyl ester, 3-methyl butanoic acid butyl ester, 2-methyl-2-hydroxy-propanoic acid ethyl ester, vinyl acetate, diethyl phthalate, 1,2-phthalic acid ester, isopentyl nitrite, 2-isohexyl sulfurous essien ester, butyl-2-ethylhexyl 1,2-phthalate, DL-alanine ethyl ester, ethyl caproate, ethyl phenylacetate, 1-hydroxy-1-cyclopropanecarboxylic acid ester); **ethers** (3-tert-butyl-4-hydroxyanisole); **heterocycles** (2,5-dimethyl pyrazine, 5-methyl-2-furanmethanol, 5-methyl-2-furancarboxaldehyde, 2,3,5-trimethyl pyrazine, 2,3,5,6-tetramethylpyrazine, benzothiazole, 2,2′,5,5′-tetramethylbiphenyl, 1,1 ′- (1-butenyl) biphenyl, 3,4-diethyl-1,1 ′- biphenyl, pterin, trigonelline); **nitrogen-containing compounds** (2-formamide (2-aminoethyl) - *N*-methoxyaziridine, L-alanine acetamide, trimethylamine *N*-oxide, methylamine, histamine, choline); **phenolic compounds** (phenol, guaiacol, 2-methoxy-4-vinylphenol, 3,5-diisopropylphenol, genistein); **Miscellaneous** (α-glucose, β-glucose, glucose-1-phosphate, soybean lecithin)	Nuclear magnetic resonance (^1^H NMR); headspace-solid phase microextraction- GC-MS (HS-SPME-GC-MS)	Yang et al., 2012, Lin et al., 2013a, Liu et al., 2018c, Gao Y. X. et al., 2020
*Bacillus pumilus*	Chicory roots fermentation	**Hydrocarbons** (hexadecane, 3,7-dimethylnonane, tetradecane, 2,2-dimethyl butane, 2,4-ditertbutyl-1,3-pentadiene, 1,4-cyclohexane); **Acids** (caproic acid, n-caprylic acid, palmitic acid, stearic acid, linoleic acid); **alcohols** (benzyl alcohol, phenethyl alcohol, phenethyl alcohol, isooctanol); **aldoketones** (phenylacetaldehyde, nonanal, decanal); **esters** (methyl palmitate, 2,6-bis (1,1-dimethyl ethyl) - 4-methylaminophenol formate, diethyl phthalate, dibutyl phthalate); **heterocycles** (trimethyl pyrazine, 2-pyrrolecarbaldehyde, γ-Hexalactone, 2-acetylpyrrole, 2-ethyl-3,5-dimethyl pyrazine, 5-methyl-1H-pyrrole-2-carbaldehyde, indole, eugenol, vanillin, 2,2′,5,5′-tetramethylbiphenyl, 3,4′-diethylbiphenyl, naphthofurans); **phenolic compounds** (o-methoxy-phenol, 4-vinylguaiacol); **nitrogen-containing compounds** (2-formamide (2-aminoethyl) - n-methoxyaziridine)	Simultaneous distillation extraction -gas chromatography-mass spectrometry (SDE/GC-MS); HS-SPME-GC-MS	Yang et al., 2012, Yang P. et al., 2019
*Bacillus amyloliquefaciens*	Soybean/wheat fermentation	**Hydrocarbons** (1-octene); **acids** (2-methylpropanoic acid, 3-methylbutanoic acid, 4-methylpentanoic acid, acetic acid, butanoic acid, 3-nitropropanoic acid, 3-methyl-butanoic acid, pentanoic acid, hexanoic acid, octanoic acid, nonanoic acid); **alcohols** (pent-1-en-3-ol, 3-methylbutan-1-ol, 2-phenylethanol, 1-octen-3-ol, 2,3-butanediol, 2-pentadecanol, 2-octanol, 2,3,4-trimethyl-3-pentanol, furfuryl alcohol, benzyl alcohol, phenylethyl alcohol, ethanol, 3-methyl-1-butanol, 2-methyl-1-butanol, 1-hexanol); **aldoketones** [(*E*)-oct-2-enal, benzaldehyde, acetaldehyde, 2-methylpropanal, 2-methylbutanal, 3-methylbutanal, butane-2,3-dione, 4-ethenylphenol, hexanal, 2-phenylacetaldehyde, heptan-2-one, octan-2-one, nonan-2-one, 2-furancarboxaldehyde, benzaldehyde, benzacetaldehyde, 2-methyl-benzaldehyde, (*E*)-11-hexadecenal, acetone, 2-propanone, 2,3-butanedione, acetoin, 1-phenyl-ethanone, dihydro-5-pentyl-2(3H)-furanone, 5-hexyldihydro-2(3H)-furanone];		
		**esters** (ethyl 2-methylpropanoate, ethyl 2-methylbutanoate, 3-methylbutyl acetate, methyl 4-methylpentanoate, ethyl hexanoate, ethyl acetate, butyl heptadecyl sulfuroate, 2-methyl-hexyl propanoate, 2-methyl-2-methylpropyl propanoate, 2-octyl benzoate, ethyl hexadecanoate, methyl 9,12-octadecadienoate, ethyl (9Z,12Z)-9,12-octadecadienoate); **heterocycles** (2-butylfuran, 2-pentylfuran, 2-ethylfuran, maltol, 2,5-dimethylpyrazine, trimethyl pyrazine, 2,3,5,7-tetramethyl pyrazine, 2-pentyl-furan, 2,3-dihydro-benzofuran); **phenolic compounds** (phenol, 2-methyl-phenol, 2-methoxy-phenol, 4-ethyl-2-methoxy-phenol, 2-methyl-4-vinylphenol, 2-methoxy-4-vinylphenol, 2,6-dimethoxy-phenol, 4-vinylphenol); **sulfur compounds** (methanesulfonic anhydride, 1-docosanethiol, *N*-ethyl-hydrazinecarbothioamide); miscellaneous (1,2-dimethoxy-benzene, 6-methyl-2-phenylindole)	HS-SPME-GC-MS	Hong et al., 2012, Seo et al., 2018
*Bacillus atrophaeus*	sorghum fermentation	**alcohols** (2,3-butanediol); **aldoketones** (acetoin, butane-2,3-dione, 2-heptanone); **phenolic compounds** (guaiacol)	GC-MS	Liu et al., 2018c
*Bacillus atrophaeus*	LB broth medium	**Hydrocarbons** (1-tetradecane, hexadecane, 1-octadecene, octadecane, 1-non-adecene, docosane, heptadecane, tetrapentacontane, eicosane); **acids** (hexadecanoic acid, phthalic acid, chloroacetic acid, *cis-*3-octyloxiraneoctanoic acid, hexanedioic acid, propanoic acid); **alcohols** (1-tetradecanol, 1-hexadecanol, cyclobutanol, erythritol, isophytol, L-alaninol); **aldoketones** (*O*-anisaldehyde, 3-buten-2-one); **esters** (methyl stearate); **heterocycles** (decamethyl-ccyclopentasiloxane, dodecamethyl-cyclohexasiloxane, hexadecamethyl-cyclooctasiloxane, octadecamethyl-cyclononasiloxane, 2-methylaminomethyl-1,3-dioxolane); **nitrogen-containing compounds** (dimethylamine, 2-octanamine, 2-hexanamine, propenamide, benzamide, *N,N*-dimethyl-methanesulfonamide, 2,3-dimethoxybenzamide, dimethyl methanesulfonamide); **phenolic compounds** (phenol); **Miscellaneous** (cyclopropyl carbinol, silane)	GC-MS	Zhang X. et al., 2013, Rajaofera et al., 2019
*Bacillus fusiformis*				-
*Bacillus badius*				-
*Bacillus bataviensis*	Lycium chinense Miller (Goji) fermentation	**Hydrocarbons** [(*Z*)-3-ethyl-2-methyl-1, 3-hexadiene, (*E, E*)-2, 6-Non-adienal, naphthalene]; **acids** (formic acid, acetic acid, hexanoic acid, octanoic acid, n-hexadecanoic acid); **alcohols** (1-hexanol, 1-octen-3-ol, benzyl alcohol, 3, 7-dimethyl-1, 6-octadien-3-ol, phenylethyl alcohol, (*E*) -2-nonen-1-ol, geraniol); **aldoketones** (hexanal, (*E*) -2-heptenal, benzaldehyde, (*E, E*) -2, 4-heptadienal, benzeneacetaldehyde, (*E*) -2-non-enal, 2, 4-dimethyl-benzaldehyde, 2, 6, 6-trimethyl-1, 3-cyclohexadiene-1-carboxaldehyde, acetoin, 6-methyl-5-hepten-2-one, 6-methyl-3,5-heptadiene-2-one, 2-n-hexylcyclopentanone, *(E*)-6, 10-dimethyl-5, 9-undecadien-2-one, *trans*-. beta. -Ionone, 5, 6, 7, 7a-tetrahydro-4, 4, 7a-trimethyl-2(4H)-benzofuranone); **ethers** (sulfurous acid-nonyl pentyl ester, Phthalic acid, isobutyl nonyl ester, hexadecanoic acid-methyl ester, hexadecanoic acid-ethyl ester); **heterocycles** (2-pentyl-furan); **nitrogen-containing compounds** (*N*, *N*-dibutyl-formamide); **phenolic compounds** (2-methoxy-phenol, 2, 3, 5-trimethyl-phenol, 2-methoxy-4-vinylphenol)	HS-SPME-GC-MS	Liu Y. et al., 2020
*Bacillus brevis*				
*Bacillus circulans*	Vinegar brewing mass fermentation	Six of high content amino acids were detected: arginine, alanine, glutamic acid, threonine, valine and leuconic acid; thirty-five volatile compounds were detected (data not shown), the high content compounds were as follows: **acids** (ethyl palmitate, iso-valerate, caprylic acid, hexanoic acid); **alcohols** (phenyl ethanol); **esters** (phenyl ethyl acetate, ethyl palmitate)	High performance liquid chromatography (HPLC)/HS-SPME-GC-MS	Wang et al., 2016
*Bacillus cereus*	Sorghums/corn fermentation	**Hydrocarbons** (3,7-dimethylnonane, 3-tetradecene, tetradecane, 6-methyl-1,3,5-cycloheptene, 1,3-dimethylnaphthalene, pentadecane, 1-pentadecyne); **acids** (isobutyric acid, acetic acid); **alcohols** (phenethyl alcohol, 2,3-butanediol, glycerol); **aldoketones** (acetoin furaldehyde); **esters** (DL-alanine ethyl ester, ethyl caproate, ethyl phenylacetate, 2-phenylethyl acetate, 1-hydroxy-1-cyclopropanecarboxylic acid ester); **heterocycles** (benzothiazole, 2-Methoxy-4-vinylphenol, 2′,5,5′-tetramethylbiphenyl, 2,3-dimethylpyrazine, 2,3,5-trimethylpyrazine, 2,3,5,6-tetramethylpyrazine); **phenolic compounds** (guaiacol)	HS-SPME-GC-MS	Yang et al., 2012, Cheng G. et al., 2021
*Bacillus coagulans*				-
*Bacillus endophyticus*				-
*Bacillus licheniformis*	Sorghum/corn/Lycium chinense Miller (Goji) fermentation	**Hydrocarbons** (n-hexadecane, 3,7-dimethylnonane, 3-tetradecane, 3,8-dimethylundecanone, nonadecane, (*Z*) -3-ethyl-2-methyl-1, 3-hexadiene, (*E, E*) -2, 6-non-adienal, naphthalene); **acids** (acetic acid, 2-methyl propanoic acid, propanoic acid, butanoic acid, 3-methyl butanoic acid, 2-methyl butanoic acid, 3-methyl-2-butenoic acid, 4-methyl-3-pentenoic acid, formic acid, hexanoic acid, octanoic acid, n-hexadecanoic acid); **alcohols** (ethanol, isopropyl alcohol, 2-methyl propanol, 3-methyl-butanol, butanol, pentanol, 1, 3-butanediol, isoborneol, borneol, α-terpineol, 2-butanol, β-phenylethanol, 2, 3-butanediol, cedrol, furfuryl alcohol, benzyl alcohol, 3, 7-dimethyl-1, 6-octadien-3-ol, phenylethyl alcohol, (*E*)-2-nonen-1-ol); **aldoketones** (2, 3-butanedione, 2-pentanone, 3-methy1-2-pentanone, acetoin, 4-hydroxy -2 -butanone, 2-heptanone, 5-hydroxy-4-octanone, benzeneace taldehyde, 2-pentanone, hexanal, (*E*)-2-heptenal, (*E, E*) -2, 4-heptadienal, benzaldehyde, benzeneacetaldehyde, (*E*)-2-non-enal, (*E, E*) -2, 4-decadienal, 6-methyl-5-hepten-2-one, 1-(1H-pyrrol-2-yl)-ethanone, 6-methyl-3, 5-heptadiene-2-one, (*E*)-6,10-dimethyl-5,9-undecadien-2-one, *trans*-.beta.-ionone, 5, 6, 7, 7a-tetrahydro-4, 4, 7a-trimethyl-2(4H)-benzofuranone); **esters** (2-methyl-2-hydroxy propanoic acid ethyl ester, butanoic acid butyl ester, 3-methyl butanoic acid butyl ester, vinyl acetate, dibutyl phthalate, 2-ethyl-isohexyl-sulfurous acid hexyl ester, ethyl caproate, 1-hydroxy-1-cyclopropanecarboxylic acid ester, benzoic acid 2-ethylhexyl ester, 6-ethyl-3-octyl butyl phthalate, *N*-methoxy-phenyl-oxime, phthalic acid-isobutyl nonyl ester, hexadecanoic acid-methyl ester, hexadecanoic acid-ethyl ester); **heterocycles** (2-methylpyridine, 2,3, 5-methyl pyrazine, 2, 5-dimethylpyrazine, 5-methyl-2-furanme thanol, 5-methyl-2-furancarboxaldehyde, 2, 3, 5, 6-tetramethyl pyrazine, benzothiazole, 2,2′,5,5′-tetramethylbiphenyl, 3,4-diethyl-1,1′- biphenyl, 2-pentyl-furan); **nitrogen-containing compounds** (L-alanine methylamide, L-alanine acetamide, 2-methyl-1-(2-nitrophenyl) -3 -(phenylmethoxy) benzene, *N*, *N*-dibutyl-formamide); **phenolic compound** (guaiacol, phenol, 2-methoxyphenol, 2, 3, 5-trimethyl-phenol, 2-methoxy-phenol)	HS-SPME-GC-MS	Yang et al., 2012, Lin et al., 2013b, Liu et al., 2018c, Liu Y. et al., 2020
*Bacillus megaterium*	Sorghum fermentation	**Alcohols (**isopentyl alcohol)**; aldoketones** (acetoin); **phenolic compounds** (guaiacol)	GC-MS	Liu et al., 2018c
*Bacillus niabensis*				-
*Bacillus pulvifaciens*				-
*Bacillus simplex*				-
*Bacillus sphaericus*				-
*Bacillus velezensis*	Minced fish fermentation	**Hydrocarbons** (3,5,5-trimethyl-1-hexene); **acids** (3-methylbutanoic acid, 2-methylbutyric acid, caproic acid); **alcohols** (1-penten-3-ol, *cis-*2-penten-1-ol, 1-hexanol, 1-octen-3-ol, 2,7-octadienol, 1-nonen-3-ol, phenylethyl alcohol); **aldoketones** (isobutyraldehyde, isovaleraldehyde, 2-methylbutyraldehyde, (*E*) 2-methyl-2-butenal, caproaldehyde, 2-ethyl-2-butenal, *trans*-2-hexenal, 2-ethyl-2-hexenal, n-capryl(ic) aldehyde, benzaldehyde, (*E)*-4-oxohexyl-2-enoaldehyde, decanal, 4-ethylbenzaldehyde, 2-butanone, 3-pentanone, 5-methyl-2-hexanone, 3-hepten-2-one, 2,3-dimethyl-2-cyclopentene-1-one, (E, *E*)-3,5-octadiene-2-one, 2,3-dimethyl-2-cyclopentene-1-one); **esters** (hexyl formate, γ-heptalactone); **heterocycles** (2-ethyl furan, *cis-*2-(2-pentenyl) furan, 2-methylpyridine, 2-ethylpyridine, 2-amino-4-methylpyridine); **nitrogen-containing compounds** (trimethylamine); **phenolic compounds** (4-ethylphenol)	HS-SPME-GC-MS	Yang H. et al., 2019
*Bacillus velezensis*	MOLP medium fermentation	**Hydrocarbons** (nonane, 8-methylheptadecane); **acids** (isovaleric acid); **alcohols** (1-butanol, isoamyl alcohol, 2-ethylhexanol, 2,3-butanediol, 1-phenylethanol); **aldoketones** (benzaldehyde, butane-2,3-dione, 2-heptanone, acetoin, 2-non-anone, 2-undecanone); **esters** (butyl formate); **heterocycles** (2,3-dimethylpyrazine, pyrazine, tetramethylpyrazine)	SPME-GC-MS	Calvo et al., 2020
*Brevibacillus brevis*	fermentation medium	**acids** (filicinic acid, diethyldithiophosphinic acid, 2-acetylamino-3-cyano-propionic acid); **alcohols** (3-octanol); **aldoketones** (6-dimethyl-6-nitro-2-hepten-4-one); **esters** (ethylparaben, dibutyl phthalate, 1,2-benzenedicarboxylic acid, butyl-2-methylpropyl ester, 1,2-benzenedicarboxylic acid-disooctyl ester, mono (2-ethylhexyl) phthalate, propylparaben, benzoic acid-3-amino-4-propoxy-2-(diethylamino)-ethyl ester); **heterocycles** (3-benzyl-hexahydropyrrole [1,2-a] pyrazine-1,4-dione, hexahydro pyrrole [1,2-a] pyrazine-1,4-dione, maltol, 3-isobutyl-hexahydropyrrole [1,2-a] pyrazine-1,4-dione, 3,6-diisobutyl-2,5-piperazine dione, 5-nitroso-2, 4, 6-triaminopyrimidine, dihydro-4,4-dimethyl-2,3-furandione); **nitrogen-containing compounds** (dihydroergotoxine); **phenolic compounds** (phenol, 3,5-dimethoxyphenol); **miscellaneous** [(4-acetylphenyl)phenylmethane, methoxyphenyl oxime]	GC-MS	Che et al., 2012
*Brevibacillus brevis*	Sorghum fermentation	**Alcohols** (2,3-butanediol, 3-pentanol, isopropanol); **aldoketones** (acetoin, butane-2,3-dione, 2-heptanone)	SPME-GC-MS	Liu et al., 2017c
*Lysinibacillus sphaerieus*	Sorghum fermentation	**Alcohols** (2,3-butanediol, 3-pentanol, ethanol, isobutanol, isoamyl alcohol); **aldoketones** (acetoin, butane-2,3-dione, 6-methyl-2-heptanone); **phenolic compounds** (guaiacol)	SPME-GC-MS	Liu et al., 2017c
*Lysinibacillus boronitolerans*				-
*Paenibacillus Castaneae*				-
*Paenibacillus larvae subsup. Pulvifaciens*	Sorghum fermentation	**Acids** (2-valeric acid); **alcohols** (isobutanol, isopropanol, 1-octen-3-ol); **aldoketones** (acetoin, butane-2,3-dione, 2-heptanone, 2-non-anone); **phenolic compounds** (guaiacol)	SPME-GC-MS	Liu et al., 2017c
*Paenibacillus macerans*				-
*Paenibacillus nicotianae*				-
*Rummeliibacillus*				-
*Sporolactobacillus*				-
*Virgibacillus pantothenticu*	Sorghum fermentation	**Alcohols (**isopentyl alcohol, 3-pentanol)**; aldoketones** (acetoin, butane-2,3-dione, 2-heptanone, 2-non-anone); **phenolic compounds** (guaiacol)	GC-MS	Liu et al., 2018c
*Clostridium butyricum*	Cheeses fermentation	**Acids** (acetic acid, propionic acid, butyric acid, pentanoic acid); **alcohols** (1-butanol); **aldoketones** (2-butanone, 2,3-butanedione, acetoin); **esters** (ethyl acetate, ethyl butanoate)	SPME-GC-MS	Gómez-Torres et al., 2015
*Clostridium kluyver*				-

*-, no published data. Bold indicates classification name of compound.*

*Bacillaceae* is the most abundant genus in pit mud, which plays a key role in SAB production. It is involved in the formation of major flavors in SAB, for example, ethyl caproate, ethyl butyrate, and caproic acid, which have been correlated positively with *Clostridium*, *Rummeliibacillus* in pit mud (Liu M. et al., 2020). Amylase, glycosylase and debranched enzyme could be generated by *Bacillus amylolitica*, and butyric acid, acetoin, volatile acid could be synthesized by *Clostridium* and *Bacillus* in pit mud (Liu et al., 2015; Wu et al., 2019). *Bacillaceae* can be employed as an indicator of pit mud quality: when the total number of *Bacillaceaes* in the new pit mud is at a high level, with the improvement of pit mud quality, the relative abundance of *Clostridium kluyver* and other *Clostridiums* increased significantly (Hu X. et al., 2016). The relative abundance of *Bacillus* decreased inversely in aged pit mud (Yu and Liu, 2016). By depressing the reproduction of harmful bacterial, one study revealed that *Clostridium butyricum* produced a variety of enzymes, which could decompose polysaccharides into oligosaccharides, and produce multiple secondary metabolites, such as antibacterial peptide, butyric acid, acetic acid, and vitamins, and that they could inhibit the propagation of harmful bacteria (Xu P. et al., 2019).

*Bacillaceae* in pit mud not only metabolizes caproic acid, butyric acid, acetic acid and other important aromatic components of SAB, but also could metabolize some secondary metabolites, which interact and regulate the related microorganisms in pit mud, thus affecting the aroma components of SAB.

### Bacillaceae in Zaopei

Research has shown that there are various *Bacillaceae* in *Zaopei* (Yao et al., 2010; Wang et al., 2011; Hu et al., 2021a). 16S rDNA analysis found multiple microbe species in *Zaopei*, *Bacillus*, and *lactobacillu*s (Liu F. et al., 2020; Hu et al., 2021a; Li and Qiu, 2021). The distribution species of microbes in *Zaopei* may be related to the specific environment in a mud pit, such as sealed hypoxia, low pH, high content of ethanol, so the growth of *Bacillaceae* is suited to this specific micro-ecological environment. As fermentation progresses, the relative abundance of *Bacillaceae* increases (Chen et al., 2010). Zhang et al. (2010) investigated bacteria flora from *Zaopei* and pit mud in Guizhou province, and 477 strains were isolated, *Bacillus Licheniformis* was identified as the dominant group of *Bacillus*, accounting for 32.96% of the total number of *Bacillus*, meanwhile, a small number of uncertain strains of *Brevi Bacillus* were isolated from *Zaopei*.

*Bacillaceae* are essential for *Zaopei*. They function by firstly secreting multiple hydrolases in the early fermentation phase, characteristics that ensure full utilization of *Daqu* and *Zaopei* (Li and Qiu, 2021). For example, organic matter such as starch, protein, and purine can be decomposed by *Clostridium* (Fan et al., 2007; Hahnke et al., 2014; Zhang et al., 2014; Yang and Chen, 2021). As they are involved in the formation of flavor matter in SAL, *Bacillus* and *Clostridium* are responsible for the production of ethyl hexanoate, caproic acid, butyric acid, lactic acid, benzaldehyde, alcohols, fatty acids, phenol, and other compounds (Hu et al., 2015; Liu et al., 2018a; Chai et al., 2019; Table 2). In summary, *Bacillaceae* of *Zaopei* was very similar to pit mud, because they were in the same container during the braving process of SAB.

### Bacillaceae in Huangshui

*Huangshui* was the main byproduct in SAB production, and presents a dark brown viscous liquid form that seeps into the bottom of the pit cellar during the fermentation process (Feng et al., 2017). Studies have shown that various organic matter can be produced by the microbes in *Huangshui* (e.g., alcohols, aldehydes, organic acid, esters, starch, reducing sugars, yeast autolysis, and other nutrients) (Zou et al., 2018; He F. et al., 2020). The microorganisms mainly consist of bacteria in *Huangshui*, among which the dominant genus was *Clostridium, Lactobacillus, and Serratia* (Li et al., 2015, 2020; Xie et al., 2020).

*Bacillaceae* are involved in *Huangshui* formation, which plays a key role in metabolizing nutrients, and could produce flavor components such as esters, acerbity, ketone, aldehyde (Li et al., 2015). Unfortunately, up to now, only a few studies have reported on the function of bacterial in *Huangshui*, especially for investigating *Bacillaceae*. At present, *Clostridium* spp. and *Bacillus* spp. had found the capacity of cellulose degradation in *Huangshui* (Desvaux et al., 2000; Sasaki et al., 2012). Six strains of *Bacillus* were isolated from *Huangshui*, which could produce cellulase, including *Bacillus cereus, Bacillus circulans, Bacillus megaterium, Bacillus endophyticus, Bacillus simplex*, and *Bacillus bataviensis*, separately (Zeng et al., 2016). During the SAB production, cellulase producing bacteria could degrade the cellulose in mixed raw materials, and release the starch inside it, which is conducive to the action of the saccharifying enzyme, thus furthering the utilization rate of raw materials, improving the fermentation rate, and shortening the fermentation time (Hu D. et al., 2016). However, the structure and reaction mechanism of *Bacillaceae* cellulase are still unclear in *Huangshui*. Recently, a redundancy analysis of the microbe community structure and aroma components showed that SAB aroma components were positively correlated with Clostridia, but negatively correlated with Bacilli in *Huangshui*. Meanwhile, acidity was positively correlated with Bacilli and negatively correlated with Clostridia (Gao Z. et al., 2020).

## Conclusion and Perspectives

At present, little information is available on the specific metabolite of each *Bacillaceae* species. This information is crucial for applying *Bacillaceae* in SAB making. Other approaches to food fermentation by the bioaugmentation of special *Bacillaceae* may provide references for future research (Table 2). Some *Bacillus* species associated with SAB making showed the ability to produce various substances, such as hydrocarbons, organic acids, alcohols, aldoketones, esters, ethers, heterocycles, nitrogen-containing compounds, and phenolic compounds. All the metabolites listed above were identified by simulated fermentation (Table 2). Unfortunately, we still lack information about other *Bacillus* species, *Clostridium* species and metabolites, and further work is required.

In the process of making SAB, various microorganisms (associated with microbial proliferation and metabolism) interact, which contributes to the diversity of SAB flavor. The synergistic effect of diverse esters has been detected between pit mud microbes and *Daqu* microbes, for example, volatile acids and alcohols were provided by pit mud. Subsequently, esterifying reactions are achieved in SAB making by *Daqu* (Gao et al., 2021). As one of the main brewing microorganisms, *Bacillaceae* interact with other microorganisms, and this interaction effect has been studied by the method of single-strain bioaugmentation. However, there are at present few studies on the interaction among SAB brewing microorganisms. Previous research suggests that bioaugmentation of *Bacillus velezensis* and *Bacillus subtilis* in *Daqu*, which alter the microbial community and improve the flavor character of *Daqu* (He et al., 2019a). Bioaugmentation with *Hydrogenispora* could affect the abundance of *Clostridium* in pit mud (Chai et al., 2019). The study found that lactic acids were cut down by bioaugmentation with *Bacillus* during the SAB fermentation and the cause was *Bacillus* was negatively related to other bacterial species (Wang et al., 2017).

There are three main problems in the study of SAB *Bacillaceae* function: firstly, we lack studies on the interaction among SAB brewing microorganisms, to better understand the relationship between metabolic mechanism and the flavor production of brewing microorganisms, the interaction between *Bacillaceae* and other microbes should be further explored. Secondly, owing to the particularity of the mud pit environment, the anaerobic strains of *Bacillaceae* are difficult to isolate and culture from pit mud and *Huangshui*, so that functional properties of some *Bacillaceae* are difficult to determine. Thirdly, there are few studies on the isolation, culture, and flavor characteristics of a single strain of SAB *Bacillaceae*, especially the effect on solid-state fermentation. Fourthly, due to the different methods of metabolite detection on strains, as a result, different compounds were detected in different experiments with the same strain. Encouragingly, new technology can help solve the challenge, for instance, multi-omics approaches (metagenomes, metatranscriptomes, metaproteomes, and metabolomes) enable us to unravel the effects of *Bacillaceae* in the production of SAB. However, the high level of ethanol, acids, and humus in samples should be properly resolved. Isotope labeling and other biotechnology have been applied to study flavor formation pathways in *Bacillaceae* and will benefit from further exploration of the metabolic mechanism of flavor substances in SAB *Bacillaceae*. In the future, further study will promote the utilization of *Bacillaceae* in the brewing process, and improve the quality and stability of SAB.

## Author Contributions

WT, PH, and YY carried out the initial literature research and manuscript writing. ZQ, DH, and HL helped to provide expertise and insight relating to *Baijiu* microbiology. WT and XF revised the manuscript. All authors read and approved the final manuscript.

## Conflict of Interest

WT and ZQ were employed by the company Wuliangye Yibin Co. Ltd. The remaining authors declare that the research was conducted in the absence of any commercial or financial relationships that could be construed as a potential conflict of interest.

## Publisher’s Note

All claims expressed in this article are solely those of the authors and do not necessarily represent those of their affiliated organizations, or those of the publisher, the editors and the reviewers. Any product that may be evaluated in this article, or claim that may be made by its manufacturer, is not guaranteed or endorsed by the publisher.

## References

[B1] Beaumont M. (2002). Flavouring composition prepared by fermentation with *Bacillus* spp. *Int. J. Food Microbiol.* 75 189–196. 10.1016/S0168-1605(01)00706-1 12036142

[B2] Calvo H. Mendiara I. Arias E. Gracia A. P. Blanco D. Venturini M. E. (2020). Antifungal activity of the volatile organic compounds produced by *Bacillus velezensis* strains against postharvest fungal pathogens. *Postharvest Biol. Technol.* 166:111208. 10.1016/j.postharvbio.2020.111208

[B3] Chai L. Xu P. Qian W. Zhang X. Ma J. Lu Z. (2019). Profiling the Clostridia with butyrate-producing potential in the mud of Chinese liquor fermentation cellar. *Int. J. Food Microbiol.* 297 41–50. 10.1016/j.ijfoodmicro.2019.02.023 30878841

[B4] Che J. Chen Z. Shi H. Liu B. (2012). Functional components in *Brevibacilus brevis* FJAT-0809-GLX determined by GC/MSD. *Fujian J. Agric. Sci.* 27 1106–1111. 10.19303/j.issn.1008-0384.2012.10.016

[B5] Chen M. Yang D. Qian Z. Zhen D. Peng D. Fang S. (2010). Analysis of microorganisms and physicochemical properties in *Zaopei* during the fermentation of Chinese zhijiang-flavor liquor. *Afr. J. Biotechnol.* 9 3874–3882.

[B6] Cheng G. Zheng Z. Wei C. Huang Z. (2021). Mixed fermentation effect of *Bacillus cereus* Frankland and *Saccharomyces cerevisiae*. *Liquor Mak. Sci. Technol.* 321, 53–59. 10.13746/j.njkj.2020203

[B7] Cheng T. Yin X. Xia Y. Su J. (2021). Study on the identification of ethyl caproate doped in Wuliangye strong aromatic liquor produced by solid-state fermentation process. *Packag. Food Mach.* 39 46–51. 10.3969/j.issn.1005-1295.2021.03.008

[B8] Deng L. Mao X. Liu D. Ning X. Shen Y. Chen B. (2020). Comparative analysis of physicochemical properties and microbial composition in high-temperature Daqu with different colors. *Front. Microbiol.* 11:3010. 10.3389/fmicb.2020.588117 33329462 PMC7732550

[B9] Desvaux M. Guedon E. Petitdemange H. (2000). Cellulose catabolism by *Clostridium cellulolyticum* growing in batch culture on defined medium. *Appl. Environ. Microbiol.* 66 2461–2470. 10.1128/AEM.66.6.2461-2470.2000 10831425 PMC110559

[B10] Ding X. Wu C. Huang J. Zhou R. (2015). Interphase microbial community characteristics in the fermentation cellar of Chinese Luzhou-flavor liquor determined by PLFA and DGGE profiles. *Food Res. Int.* 72 16–24. 10.1016/j.foodres.2015.03.018

[B11] Ding X. Wu C. Zhang L. Zheng J. Zhou R. (2014). Characterization of eubacterial and archaeal community diversity in the pit mud of Chinese Luzhou-flavor liquor by nested PCR–DGGE. *World J. Microbiol. Biotechnol.* 30 605–612. 10.1007/s11274-013-1472-4 24030168

[B12] Fan W. Xu Y. Zhang Y. (2007). Characterization of pyrazines in some Chinese liquors and their approximate concentrations. *J. Agric. Food Chem.* 55 9956–9962. 10.1021/jf071357q 17970591

[B13] Fan W. Zhao X. Du G. Chen J. Li J. Zheng J. (2021). Metaproteomic analysis of enzymatic composition in Baobaoqu fermentation starter for Wuliangye baijiu. *Int. J. Food Sci. Technol.* 56 4170–4181. 10.1111/ijfs.15047

[B14] Feng X. Deng J. Xie J. Wei C. Luo H. Huang Z. (2017). Brief analysis on current situation of comprehensive utilization of by-products yellow water from baijiu-making. *China Brew.* 36 6–9. 10.11882/j.issn.0254-5071.2017.02.002

[B15] Fu X. Liu X. Ao Z. Han G. Liu X. Qiu D. (2011). Research progress in typical fermented grains of Chinese strong aromatic spirits and its influencing factors. *J. Beijing Technol. Bus. Univ. Nat. Sci. Ed.* 6 54–57.

[B16] Gao J. Liu G. Li A. Liang C. Ren C. Xu Y. (2021). Domination of pit mud microbes in the formation of diverse flavour compounds during Chinese strong aroma-type baijiu fermentation. *LWT* 137:110442. 10.1016/j.lwt.2020.110442

[B17] Gao Y. X. Xu B. Fan H. R. Zhang M. R. Zhang L. J. Lu C. (2020). ^1^H NMR-based chemometric metabolomics characterization of soymilk fermented by *Bacillus subtilis* BSNK-5. *Food. Res. Int.* 138:109686. 10.1016/j.foodres.2020.109686 33292958

[B18] Gao Z. Wu Z. Zhang W. (2020). Effect of pit mud on bacterial community and aroma components in yellow water and their changes during the fermentation of Chinese strong-flavor liquor. *Foods* 9:372. 10.3390/foods9030372 32210161 PMC7143002

[B19] Gómez-Torres N. Garde S. Peirotén Á Ávila M. (2015). Impact of *Clostridium* spp. on cheese characteristics: microbiology, color, formation of volatile compounds and off-flavors. *Food Control* 56 186–194. 10.1016/j.foodcont.2015.03.025

[B20] Gou M. Wang H. Yuan H. Zhang W. Tang Y. Kida K. (2015). Characterization of the microbial community in three types of fermentation starters used for Chinese liquor production. *J. Inst. Brew.* 121 620–627. 10.1002/jib.272

[B21] Gou W. Tian Y. Kong X. Wu F. Fang F. (2020). Bacterial composition in pit mud of Yanghe liquor and identification of acid producing bacteria. *Microbiol. China* 47 1651–1661.

[B22] Guan T. Lin Y. Chen K. Ou M. Zhang J. (2020). Physicochemical factors affecting microbiota dynamics during traditional solid-state fermentation of Chinese strong-flavor baijiu. *Front. Microbiol.* 11:2090. 10.3389/fmicb.2020.02090 33013762 PMC7509048

[B23] Guo X. Fan E. Ma B. Li Z. Zhang Y. Zhang Z. (2020). Research progress in functional bacteria in solid-state fermented baijiu in China. *Food Ferment. Ind.* 46 280–286. 10.13995/j.cnki.11-1802/ts.022283

[B24] Hahnke S. Striesow J. Elvert M. Mollar X. P. Klocke M. (2014). *Clostridium bornimense* sp. nov., isolated from a mesophilic, two-phase, laboratory-scale biogas reactor. *Int. J. Syst. Evol. Microbiol.* 64 2792–2797. 10.1099/ijs.0.059691-0 24860110

[B25] He F. Yang H. Zeng L. Hu H. Hu C. (2020). Production and characterization of bacterial cellulose obtained by *Gluconacetobacter xylinus* utilizing the by-products from baijiu production. *Bioprocess Biosyst. Eng.* 43 927–936. 10.1007/s00449-020-02289-6 31997008

[B26] He G. Huang J. Wu C. Jin Y. Zhou R. (2020). Bioturbation effect of fortified Daqu on microbial community and flavor metabolite in Chinese strong-flavor liquor brewing microecosystem. *Food Res. Int.* 129:108851. 10.1016/j.foodres.2019.108851 32036891

[B27] He G. Huang J. Zhou R. Wu C. Jin Y. (2019b). Effect of fortified Daqu on the microbial community and flavor in Chinese strong-flavor liquor brewing process. *Front. Microbiol.* 10:56. 10.3389/fmicb.2019.00056 30761106 PMC6361764

[B28] He G. Dong Y. Huang J. Wang X. Zhang S. Wu C. (2019a). Alteration of microbial community for improving flavor character of Daqu by inoculation with *Bacillus velezensis* and *Bacillus subtilis*. *LWT* 111 1–8. 10.1016/j.lwt.2019.04.098

[B29] Hong Y. Jung H.-J. Kim H.-Y. (2012). Aroma characteristics of fermented Korean soybean paste (Doenjang) produced by *Bacillus amyloliquefaciens*. *Food Sci. Biotechnol.* 21 1163–1172. 10.1007/s10068-012-0152-8

[B30] Hu D. Ju X. Li L. Hu C. Yan L. Wu T. (2016). Improved *in situ* saccharification of cellulose pretreated by dimethyl sulfoxide/ionic liquid using cellulase from a newly isolated *Paenibacillus* sp. LLZ1. *Bioresour. Technol.* 201 8–14. 10.1016/j.biortech.2015.11.039 26618784

[B31] Hu X. Du H. Ren C. Xu Y. (2016). Illuminating anaerobic microbial community and cooccurrence patterns across a quality gradient in Chinese liquor fermentation pit muds. *Appl. Environ. Microbiol.* 82 2506–2515. 10.1128/AEM.03409-15 26896127 PMC4959489

[B32] Hu X. Du H. Xu Y. (2015). Identification and quantification of the caproic acid-producing bacterium *Clostridium kluyveri* in the fermentation of pit mud used for Chinese strong-aroma type liquor production. *Int. J. Food Microbiol.* 214 116–122. 10.1016/j.ijfoodmicro.2015.07.032 26267890

[B33] Hu X. Tian R. Wang K. Cao Z. Yan P. Li F. (2021b). The prokaryotic community, physicochemical properties and flavors dynamics and their correlations in fermented grains for Chinese strong-flavor baijiu production. *Food Res. Int.* 148:110626. 10.1016/j.foodres.2021.110626 34507770

[B34] Hu X. Tian R. Li B. Zhang Y. Chi L. He P. (2021a). Analysis of community structure and functional changes of active microorganisms in fermented grains for the Chinese strong-flavor baijiu production based on metatranscriptomics technology. *Food Sci.* 42, 1–12. 10.7506/spkx1002-6630-20210324-293

[B35] Hu X. Wang K. Chen M. Fan J. Han S. Hou J. (2020). Profiling the composition and metabolic activities of microbial community in fermented grain for the Chinese strong-flavor baijiu production by using the metatranscriptome, high-throughput 16S rRNA and ITS gene sequencings. *Food Res. Int.* 138:109765. 10.1016/j.foodres.2020.109765 33292946

[B36] Li K. Fan Z. Wang J. Lin K. Xiang W. (2015). Microbial diversity in fermented yellow water of traditional intense flavor liquor. *J. Food Sci. Biotechnol.* 34 1155–1161. 10.3969/j.issn.1673-1689.2015.11.006

[B37] Li X. Zhang J. Yan J. Xue D. Chen M. Fang S. (2014). Dynamic analysis of microbial communities in the fermenting process of Daohuaxiang liquor by use of wrapped starter. *Liquor Mak. Sci. Technol.* 235, 16–20. 10.13746/j.njkj.2014.01.007

[B38] Li Y. Yuan S. Yong X. Liu J. (2020). Research progress on small peptides in Chinese baijiu. *J. Funct. Foods* 72:104081. 10.1016/j.jff.2020.104081

[B39] Li Z. Qiu L. (2021). Changes of prokaryotic community succession process of fermented grains during strong-flavor baijiu fermentation. *J. Shandong Inst. Light Ind. Nat. Sci. Ed.* 35 26–31. 10.16442/j.cnki.qlgydxxb.2021.04.005

[B40] Liang H. Li W. Luo Q. Liu C. Wu Z. Zhang W. (2015). Analysis of the bacterial community in aged and aging pit mud of Chinese Luzhou-flavour liquor by combined PCR-DGGE and quantitative PCR assay. *J. Sci. Food Agric.* 95 2729–2735. 10.1002/jsfa.7013 25418958

[B41] Liang H. Xu C. Tang W. Ren Y. Zhu I. (2021). Microbial community structure and diversity in Luzhou-flavor liquor cellar mud. *Mod. Agric. Sci. Technol.* 24, 203–206+220. 10.3969/j.issn.1007-5739.2021.03.078

[B42] Liang J. Li A. Li L. He H. Tang Y. Wu W. (2017). Separation, identification of microorganisms in GuJing liquor Daqu using biolog-ECO method. *Liquor Mak.* 44 28–32.

[B43] Lin Q. Dong S. Fu Q. Hu C. Pan Q. Zhou G. (2013a). Isolation of aroma-producing *Bacillus subtilis* and analysis of its fermentation metabolites. *Liquor Mak. Sci. Technol.* 11 30–32. 10.13746/j.njkj.2013.11.027

[B44] Lin Q. Xiao Z. Fu Q. Fang X. Mo L. Pan Q. (2013b). Isolation of aroma-producing *Bacillus licheniformis* and analysis of its fermentation metabolites. *Liquor Mak. Sci. Technol.* 223, 49–52. 10.13746/j.njkj.2013.12.020

[B45] Liu F. Zhou X. Chen X. Chen J. Du G. Fang F. (2018b). Microbial community of fermented grains and its correlation with organic acids biosynthesis during Yanghe strong-aroma liquor manufacturing process. *Acta Microbiol. Sin.* 12 2087–2098. 10.13343/j.cnki.wsxb.20170616

[B46] Liu Y. Wang Y. Li Y. Wang W. Liu M. Wu S. (2018c). The study of microorganisms in pit mud by Agilent GC and GC-MS. *Environ. Chem.* 37 902–905.

[B47] Liu F. Qiu Y. Zhou X. Chen X. Li Z. Chen J. (2018a). The correlation between organic acid producing bacteria and organic acids biosynthesis in fermented grains of Yanghe strong-aroma spirit. *Food Ferment. Ind.* 44 22–29. 10.13995/j.cnki.11-1802/ts.017898

[B48] Liu F. Yang K. Zhang J. Qiao Z. An M. Zheng J. (2020). Research progress in microbial diversity of fermented grains of baijiu. *Liquor Mak. Sci. Technol.* 307, 89–97. 10.13746/j.njkj.2020060

[B49] Liu M. Tang Y. Guo X. Ke Z. Penttinen P. Tian X. (2020). Structural and functional changes in prokaryotic communities in artificial pit mud during Chinese baijiu production. *mSystems* 5:e00829-19. 10.1128/mSystems.00829-19 32209718 PMC7093824

[B50] Liu M. Tang Y. Zhao K. Gu Y. Ren D. Yao W. (2017b). Recent advances in research on the community, isolation, and application of microbes in the pit mud used in manufacture of Chinese strong-flavor baijiu. *Microbiol. China* 44 1222–1229. 10.13344/j.microbiol.china.160559

[B51] Liu Y. Wang Y. Wang W. Li Y. Wu S. Liu M. (2017c). Screening, identification and metabolites analyses of *Bacillus* in pit mud of Luzhou-flavor baijiu. *China Brew.* 36 76–79. 10.11882/j.issn.0254-5071.2017.07.017

[B52] Liu M. Tang Y. Guo X. Zhao K. Tian X. Liu Y. (2017a). Deep sequencing reveals high bacterial diversity and phylogenetic novelty in pit mud from Luzhou Laojiao cellars for Chinese strong-flavor baijiu. *Food Res. Int.* 102 68–76. 10.1016/j.foodres.2017.09.075 29196000

[B53] Liu M. Zhao K. Tang Y. Ren D. Yao W. Tian X. (2015). Analysis of *Clostridium* cluster I community diversity in pit mud used in manufacture of Chinese Luzhou-flavor liquor. *Food Sci. Biotechnol.* 24 995–1000. 10.1007/s10068-015-0127-7

[B54] Liu S. Li L. Li K. Jia B. Zhong X. Che Z. (2013). Spatial heterogeneity of prokaryotic microbial communities in Luzhou-flavor liquor pit mud. *Food Sci.* 34 221–226. 10.7506/spkx1002-6630-201321045

[B55] Liu Y. Cheng H. Ye X. Liu H. Fang H. (2020). Changes of bioactive compounds and volatile compounds contents in goji juice fermented by different probiotics. *Acta Agric. Univ. Zhejiangensis* 32:499. 10.3969/j.issn.1004-1524.2020.03.16

[B56] Luo J. Zhu S. Wang L. He J. Ouyang L. Zhou J. (2020). Research progress on the composition of brewing microorganisms and flavor substances in strong-flavor baijiu. *China Brew.* 39 1–6. 10.11882/j.issn.0254-5071.2020.04.001

[B57] Ma J. Lu Z. Zhang X. Wang S. Shen C. Shi J. (2016). Role of *Clostridia* community in fermented grains during Luzhou-flavor liquor production. *Microbiol. China* 43 2322–2329. 10.13344/j.microbiol.china.160110

[B58] Qian W. Lu Z. Chai L. Zhang X. Li Q. Wang S. (2021). Cooperation within the microbial consortia of fermented grains and pit mud drives organic acid synthesis in strong-flavor baijiu production. *Food Res. Int.* 147:110449. 10.1016/j.foodres.2021.110449 34399451

[B59] Rajaofera M. J. N. Wang Y. Dahar G. Y. Jin P. Fan L. Xu L. (2019). Volatile organic compounds of *Bacillus atrophaeus* HAB-5 inhibit the growth of *Colletotrichum gloeosporioides*. *Pestic. Biochem. Physiol.* 156 170–176. 10.1016/j.pestbp.2019.02.019 31027577

[B60] Sasaki D. Morita M. Sasaki K. Watanabe A. Ohmura N. (2012). Acceleration of cellulose degradation and shift of product via methanogenic co-culture of a cellulolytic bacterium with a hydrogenotrophic methanogen. *J. Biosci. Bioeng.* 114 435–439. 10.1016/j.jbiosc.2012.05.002 22652087

[B61] Seo H. S. Lee S. Singh D. Park M. K. Kim Y.-S. Shin H. W. (2018). Evaluating the headspace volatolome, primary metabolites, and aroma characteristics of koji fermented with *Bacillus amyloliquefaciens* and *Aspergillus oryzae*. *J. Microbiol. Biotechnol.* 28 1260–1269. 10.4014/jmb.1804.04017 30301311

[B62] Shi X. Xu Y. Cui F. Zhong Y. Xie Y. Xie X. (2012). Study on the application of *B. subtilis* in the production of liquor. *Liquor Mak. Sci. Technol.* 212 49–53. 10.13746/j.njkj.2012.02.016

[B63] Tao Y. Li J. Rui J. Xu Z. Zhou Y. Hu X. (2014). Prokaryotic communities in pit mud from different-aged cellars used for the production of Chinese strong-flavored liquor. *Appl. Environ. Microbiol.* 80 2254–2260. 10.1128/AEM.04070-13 24487528 PMC3993157

[B64] Wang C. Chen Q. Wang Q. Li C. Leng Y. Li S. (2014). Long-term batch brewing accumulates adaptive microbes, which comprehensively produce more flavorful Chinese liquors. *Food Res. Int.* 62 894–901. 10.1016/j.foodres.2014.05.017

[B65] Wang J. Wan Z. Li L. Li X. Chen M. (2010). Study on the change of microflora in pit mud of Daohuaxiang. *Liquor Mak. Sci. Technol.* 11 36–39. 10.13746/j.njkj.2010.11.009

[B66] Wang S. Yan S. Jin X. (2021). Dynamic changes of bacteria and fungi in strong-flavor Daqu during fermentation. *Liquor Mak.* 48 29–34.

[B67] Wang T. Zhao D. Tian S. You L. Wang S. Feng R. (2011). Phylogenetic diversity of cultivable bacteria during the brewing process of the luzhou-flavor liquor in Yibin, Sichuan province, China. *Acta Microbiol. Sin.* 51 1351–1357. 10.13343/j.cnki.wsxb.2011.10.006 22233056

[B68] Wang W. Wang Y. Wu S. Liu M. Deng J. Li Y. (2018). Isolation and identification of three anaerobic bacteria strains in Luzhou-flavor liquor and metabolites analysis. *Food Sci. Technol.* 43 15–20. 10.13684/j.cnki.spkj.2018.02.003

[B69] Wang X. Du H. Xu Y. (2017). Source tracking of prokaryotic communities in fermented grain of Chinese strong-flavor liquor. *Int. J. Food Microbiol.* 244 27–35. 10.1016/j.ijfoodmicro.2016.12.018 28064120

[B70] Wang Y. Luo H. Liu Y. Wang Y. Ye G. (2015b). Separation and identification of *Bacillus* spp. from Luzhou-flavor Daqu. *J. Sichuan Univ. Sci. Eng. Nat. Sci. Ed.* 28 5–8. 10.11863/j.suse.2015.02.02

[B71] Wang Y. Li Z. Gan G. Jiang L. Yang Y. Sheng X. (2015a). Screening of acid production functional bacteria in pit mud and preliminary study on fermentation conditions. *Liquor Mak.* 42 52–56.

[B72] Wang Z. Lu Z. Zhu Q. Shi J. Xu Z. (2016). Fermentation properties of *Bacilus circulans*, a functional microbe from Zhenjiang aromatic vinegar brewing mas. *China Condiments* 41 24–28. 10.3969/j.issn.1000-9973.2016.09.006

[B73] Wu Q. Feng X. Guo W. Bao X. Ren N. (2020). Long-term medium chain carboxylic acids production from liquor-making wastewater: parameters optimization and toxicity mitigation. *Chem. Eng. J.* 388:124218. 10.1016/j.cej.2020.124218

[B74] Wu W. Li A. Tang Y. Li L. (2016). The flavor compound in the fermentation of *Bacillus* from Gujing pit mud. *Food Sci. Technol.* 41 27–30. 10.13684/j.cnki.spkj.2016.03.006

[B75] Wu W. Liu F. Fang S. Pu S. (2019). Flavor compounds produced by *Paenibacillus* from pit mud in northern Anhui. *Sci. Technol. Food Ind.* 40 274–279. 10.13386/j.issn1002-0306.2019.10.045

[B76] Xiang H. Lin Y. Guan T. Zhang J. Shang H. Zhao X. (2020). Diversities of culturable yeast and *Bacillus* and their relationship with process parameters during the production of Sichuan Luzhou-flavor Daqu. *Food Sci.* 41 196–201. 10.7506/spkx1002-6630-20181217-179

[B77] Xie J. Cheng K. Zhao D. Yang G. Qiao Z. Qiu S. (2020). *Bacillus aquiflavi* sp. nov., isolated from yellow water of strongly flavored Chinese baijiu. *Int. J. Syst. Evol. Microbiol.* 70 3406–3412. 10.1099/ijsem.0.004185 32375947

[B78] Xu P. Chai L. Qiu T. Zhang X. Lu Z. Xiao C. (2019). *Clostridium fermenticellae* sp. nov., isolated from the mud in a fermentation cellar for the production of the Chinese liquor, baijiu. *Int. J. Syst. Evol. Microbiol.* 69 859–865. 10.1099/ijsem.0.003254 30735112

[B79] Xu Y. Sun B. Fan G. Teng C. Xiong K. Zhu Y. (2017). The brewing process and microbial diversity of strong flavour Chinese spirits: a review. *J. Inst. Brew.* 123 5–12. 10.1002/jib.404

[B80] Xu Z. Tang Q. Xu Z. Liu M. Qiu X. (2019). Screening and application of special functional bacteria from aged pit mud in workshops of Jiannanchun Tianyilaohao. *Liquor Mak. Sci. Technol.* 295, 94–100. 10.13746/j.njkj.2019202

[B81] Yan Z. Zheng X. Han B. Han J. Nout M. Chen J. (2013b). Monitoring the ecology of *Bacillus* during Daqu incubation, a fermentation starter, using culture-dependent and culture-independent methods. *J. Microbiol. Biotechnol.* 23 614–622. 10.4014/jmb.1211.11065 23648849

[B82] Yan Z. Zheng X. Chen J. Han J. Han B. (2013a). Effect of different *Bacillus* strains on the profile of organic acids in a liquid culture of Daqu. *J. Inst. Brew.* 119 78–83. 10.1002/jib.58

[B83] Yang C. Liao Y. Liu J. Hu J. Hu J. Dou S. (2012). Identification of *Bacillus* from Niulanshan erguotou fermented grain and analysis of flavor compounds in the fermentation. *Sci. Technol. Food Ind.* 33 69–74. 10.13386/j.issn1002-0306.2012.09.073

[B84] Yang H. Ning Y. Wang C. Weng P. Wu Z. Zhu Y. (2019). Effects of inoculated fermentation on characters of anchovy fish sauce by *Bacillus velezensis* SW5. *J. Nucl. Agric. Sci.* 33 2013–2022.

[B85] Yang L. Chen L. (2021). Optimization of conditions for amylase and protease production of a thermotolerant *Bacillus licheniformis* strain. *Liquor Mak.* 48 49–53.

[B86] Yang P. Xu S. Duan Y. Li Y. Wu H. Wang K. (2019). Analysis of volatile components in fermentation extracts from chicory rootss fermented by *Bacillus pumilus*. *China Food Addit.* 30 93–99. 10.19804/j.issn1006-2513.2019.10.009

[B87] Yao W. Chen M. Zhen D. Guo Y. (2010). Isolation of lactate-producing microbes from fermented grains of Luzhou-flavor liquor and their effect on simulative solid-state fermentation. *Liquor Mak.* 37 37–41.

[B88] Yao W. Tang Y. Ren D. Liao J. Shen C. Xu D. (2005). Study on the differences of microbes in the different layers of Guojiao Daqu. *Liquor Mak.* 32 35–37.

[B89] Ye G. Luo H. Yang X. Li D. Wang Y. Ni B. (2013). Community structure of prokaryotes in pit mud of lu-flavor liquor from luzhou prefecture based on culture-independent approach. *J. Food Sci.* 34 194–199. 10.7506/spkx1002-6630-201317038

[B90] Yin X. Zhang S. Ao Z. Shui L. Li D. Liu Y. (2014). Effects of seasons on the fermentation of Nong-flavor liquor. *Liquor Mak. Sci. Technol.* 235, 51–54+58. 10.13746/j.njkj.2014.01.017

[B91] You J. Chen M. Fang S. Liu Q. Li J. (2009). Isolation and preliminary identification of dominsnt microbe in Daqu and mud pit of Zhijiang Daqu liquor. *Liquor Mak.* 36, 31–34.

[B92] Yu C. Liu C. (2016). Analysis of bacterial diversity in different areas of strong aromatic Chinese spirit in pit mud. *Food Res. Dev.* 37 148–151. 10.3969/j.issn.1005-6521.2016.24.035

[B93] Yue Y. Zhang W. Liu X. Hu C. Zhang S. (2007). Isolation and identification of facultative anaerobes in the pit mud of Chinese Luzhou-flavor liquor. *Microbiol. China* 34 251–255. 10.13344/j.microbiol.china.2007.02.013

[B94] Zeng L. Tan X. Yuan C. Zhang Q. Yang Y. Zhao T. (2016). Isolation and identification of cellulose-degrading strains from yellow water of baijiu (Chinese liquor) and its cellulase activity. *China Brew.* 35 59–63. 10.11882/j.issn.0254-5071.2016.11.012

[B95] Zhai L. Yu X. Feng H. Zhang C. Zhou L. Qiu S. (2020). Study on bacterial community structure in the brewing habitats of strong flavour Chinese baijiu from Yibin region. *Food Ferment. Ind.* 46 18–24. 10.13995/j.cnki.11-1802/ts.022600

[B96] Zhang C. Li J. Liu Z. Sun L. Dong X. Li R. (2019). Study of a caproic acid-producing functional strain from the pit mud of Nongxiang baijiu. *Liquor Mak. Sci. Technol.* 295, 50–53. 10.13746/j.njkj.2019171

[B97] Zhang L. Luo H. Huang D. Zhang Q. Zheng S. Tong W. (2021a). Screening of a *Bacillus* strain with high acetoin-producing ability and optimization of its fermentation conditions. *Mod. Food Sci. Technol.* 37 71–81. 10.13982/j.mfst.1673-9078.2021.5.0919

[B98] Zhang L. Ran M. Wang S. Liu G. Ma Z. Ren J. (2021b). Isolation and identification of strains with high yield of fibrinolysin from Daqu of Luzhou Laojiao. *Liquor Mak. Sci. Technol.* 319, 17–24. 10.13746/j.njkj.2021033

[B99] Zhang R. Wu Q. Xu Y. (2013). Aroma characteristics of Moutai-flavour liquor produced with *Bacillus licheniformis* by solid-state fermentation. *Lett. Appl. Microbiol.* 57 11–18. 10.1111/lam.12087 23594087

[B100] Zhang R. Wu Q. Xu Y. (2014). Lichenysin, a cyclooctapeptide occurring in Chinese liquor Jiannanchun reduced the headspace concentration of phenolic off-flavors via hydrogen-bond interactions. *J. Agric. Food Chem.* 62 8302–8307. 10.1021/jf502053g 25065507

[B101] Zhang X. Li B. Wang Y. Guo Q. Lu X. Li S. (2013). Lipopeptides, a novel protein, and volatile compounds contribute to the antifungal activity of the biocontrol agent *Bacillus atrophaeus* CAB-1. *Appl. Microbiol. Biotechnol.* 97 9525–9534. 10.1007/s00253-013-5198-x 24013222

[B102] Zhang X. Wu Z. Zhang S. Hu C. Zhang W. (2010). Phylogenetic analysis of cultured bacteria in Luzhou-flavor liquor pits in Guizhou. *Liquor Mak. Sci. Technol.* 198 23–27. 10.13746/j.njkj.2010.12.001

[B103] Zhang Y. Zhu X. Li X. Tao Y. Jia J. He X. (2017). The process-related dynamics of microbial community during a simulated fermentation of Chinese strong-flavored liquor. *BMC Microbiol.* 17:196. 10.1186/s12866-017-1106-3 28915790 PMC5603089

[B104] Zhao D. Niu G. Peng Z. Chen L. Yang R. (2009). Change in microflora and the evolution of physical and chemical factors in Wuliangye wrapped starter. *Liquor Mak. Sci. Technol.* 12 38–40. 10.13746/j.njkj.2009.12.003

[B105] Zhao H. Chang Y. Wang W. Ling H. Ping W. Zhao Z. (2012). Isolation and identification of facultative anaerobic strains with high yield of hexanoic acid from Luzhou-flavor liquor pit mud. *Food Sci.* 33 177–182.

[B106] Zheng J. Liang R. Zhang L. Wu C. Zhou R. Liao X. (2013). Characterization of microbial communities in strong aromatic liquor fermentation pit muds of different ages assessed by combined DGGE and PLFA analyses. *Food Res. Int.* 54 660–666. 10.1016/j.foodres.2013.07.058

[B107] Zheng X. Han B. (2016). Baijiu, Chinese liquor: history, classification and manufacture. *J. Ethn. Foods* 3 19–25. 10.1016/j.jef.2016.03.001

[B108] Zheng X. Tabrizi M. R. Nout M. R. Han B. (2011). Daqu—a traditional Chinese liquor fermentation starter. *J. Inst. Brew.* 117 82–90. 10.1002/j.2050-0416.2011.tb00447.x

[B109] Zhi Y. Wu Q. Du H. Xu Y. (2016). Biocontrol of geosmin-producing *Streptomyces* spp. by two *Bacillus* strains from Chinese liquor. *Int. J. Food Microbiol.* 231 1–9. 10.1016/j.ijfoodmicro.2016.04.021 27161758

[B110] Zhou P. Luo H. Huang D. Deng B. Wang Q. Huang Z. (2016). Separation and identification of thermoduric bacteria strains in medium/high temperature Daqu and the analysis of the flavor metabolites. *Sci. Technol. Food Ind.* 15 215–220. 10.13386/j.issn1002-0306.2016.24.033

[B111] Zhou R. Chen Y. Tang D. (2010). Research on microbial diversity & distribution in Yibin multiple-grains Luzhou-flavor liquor distillery. *Liquor Mak. Sci. Technol.* 5 60–64. 10.13746/j.njkj.2010.05.001

[B112] Zou W. Zhao C. Luo H. (2018). Diversity and function of microbial community in Chinese strong-flavor baijiu ecosystem: a review. *Front. Microbiol.* 9:671. 10.3389/fmicb.2018.00671 29686656 PMC5900010

